# Why Canada Is in Court to Protect Healthcare for All: Global Implications for Universal Health Coverage

**DOI:** 10.3389/frhs.2021.744105

**Published:** 2021-11-16

**Authors:** John Frank, Claudia Pagliari, Cam Donaldson, Kate E. Pickett, Karen S. Palmer

**Affiliations:** ^1^Usher Institute, University of Edinburgh, Edinburgh, United Kingdom; ^2^Yunus Centre for Social Business and Health, Glasgow Caledonian University, Glasgow, United Kingdom; ^3^Department of Health Sciences, Faculty of Science, University of York, York, United Kingdom; ^4^Faculty of Health Sciences, Simon Fraser University, Burnaby, BC, Canada

**Keywords:** sustainable development goals (SDGs), Canada health act, global health policy, health inequalities, Universal Health Coverage (UHC)

## Abstract

Countries worldwide are currently endeavoring to safeguard the long-term health of their populations through implementing Universal Health Coverage (UHC), in line with the United Nation's 2015-30 Sustainable Development Goals (SDGs). Canada has some of the world's strongest legislation supporting equitable access to care for medically necessary hospital and physician services based on need, not ability to pay. A constitutional challenge to this legislation is underway in British Columbia (BC), led by a corporate plaintiff, Cambie Surgeries Corporation (CSC). This constitutional challenge threatens to undermine the high bar for UHC protection that Canada has set for the world, with potential adverse implications for equitable international development. CSC claims that BC's healthcare law—the Medicare Protection Act (MPA)—infringes patients' rights under Canada's constitution, by essentially preventing physicians who are enrolled in BC's publicly-funded Medicare plan from providing expedited care to patients for a private fee. In September 2020, after a trial that ran for 3.5 years and included testimony by more than 100 witnesses from around the world, the court dismissed the plaintiffs' claim. Having lost their case in the Supreme Court of BC, the plaintiffs' appealed in June 2021. The appellate court's ruling and reasons for judgment are expected sometime in 2021. We consider the evidence before the court from the perspective of social epidemiology and health inequalities, demonstrating that structural features of a modern society that exacerbate inequalities, including inequitable access to healthcare, can be expected to lead to worse overall societal outcomes.

## Key Messages

- Canada has some of the world's strongest legislation to support equitable access to care for medically necessary hospital and physician services based on need, not ability to pay.
- Structural features of a modern society that exacerbate inequalities, including inequitable access to healthcare, can be expected to lead to worse overall societal outcomes.
- Societies that achieve Universal Health Coverage (UHC), with low or no fees at point of care, have taken a critical step toward safeguarding long-term population health, in line with the 2015-30 Sustainable Development Goals.
- The current constitutional challenge to British Columbia's Medicare law threatens to undermine the high bar for UHC protection that Canada has set for the world, with potential implications for equitable international development.

## Introduction

On September 10, 2020—nearly 4 years after starting of the “Cambie Trial” to determine the constitutionality of British Columbia's (BC) law protecting its universal “Medicare” system—the Supreme Court of BC handed down its 880-page Reasons for Judgment in *Cambie Surgeries Corporation v. British Columbia* (1). The plaintiffs, led by Cambie Surgeries Corporation, claim that BC's Medicare Protection Act (MPA) infringes patients' rights under the Canadian Charter of Rights and Freedoms. BC's Supreme court dismissed the plaintiffs' claim against the Attorney General of BC. It found that, on the balance of probabilities, the evidence before the court supported BC's law on grounds aligned with both equity and sustainability of its universal healthcare system.

The plaintiffs immediately launched an appeal in the BC Court of Appeal, the highest provincial court, which was heard June 14-18, 2021. The court's ruling and reasons for judgment are expected later in 2021. Meanwhile, the appellants also obtained an injunction to temporarily prevent BC's Medical Services Commission (MSC, which manages BC's single payer Medical Services Plan (MSP) on behalf of the BC Government in accordance with the MPA) from enforcing the law's “extra-billing” provisions in private surgical clinics for patients whose surgery has been scheduled beyond, or has not taken place by, the Ministry of Health's wait time benchmark, due to insufficient capacity in the public system (2). This case is expected to go all the way to the Supreme Court of Canada, settling it once and for all, but potentially jeopardizing Canada's federated (pan-provincial) system of Medicare.

An analysis of the massive body of global evidence accumulated and extensively analyzed during the trial on both sides of this debate (57,000 pages including exhibits from expert reports, affidavits, and research studies, plus 15,000 pages of transcripts, and nearly 1,400 pages of closing arguments) is beyond the scope of this article but is well-summarized in the court's Reasons for Judgment (1). The aim of our analysis is three-fold: (1) explain the origins of this Canadian legal struggle; (2) summarize the scientific evidence that supports the prohibition (or, at minimum, the strong regulation) of privately-funded care in countries with publicly-funded universal-coverage care systems; and (3) outline our perspective on the potential global implications of this trial for Universal Health Coverage (UHC) and our opinion on how this might influence international development.

## What is the Purpose of Bc's Medicare Law?

The purpose of BC's law is to preserve a publicly-managed and fiscally sustainable healthcare system, in which access to necessary medical care (mainly hospital and physician services) is based on need and not on an individual's ability to pay. “Medical necessity” is a guiding principle; the precise scope of services covered is not defined by statute or legislation. BC's law prohibits physicians enrolled in BC's single payer Medical Services Plan from charging patients who are MSP beneficiaries—nearly all residents of BC—for medically necessary services. This includes extra billing, user charges, and duplicative private insurance that covers care already included under MSP. It also prohibits, *de facto*, dual practice, such that physicians enrolled in MSP may not provide insured services to both public pay and private pay patients. Physicians in BC may, however, choose not to enroll in MSP, in which case they can charge patients directly for medically necessary care at whatever rate the market will bear, so long as they do not treat patients in hospitals or community care facilities. Although Canada's federal health legislation—the Canada Health Act (CHA) (3)—itself was not directly challenged, its principles were because they parallel those of BC's law. Thus, the Attorney General of Canada intervened in the trial to support BC's legislation and the principles of the Canada Health Act embedded in it.

## What is the Global Relevance of *Cambie Surgeries Corporation v. British Columbia*?

Many nations are moving steadily toward Universal Health Coverage (UHC), one of the United Nation's Sustainable Development Goals. Initially, this push arose from within the World Health Organization (4–6). More recently, key international development authorities, such as the World Bank, have also supported UHC (7). This is rather remarkable, given that some of them had previously advised global nations to pursue policies antithetical to the spirit of UHC, such as user fees at point of care (8). UHC is now seen as a critical policy plank in moving countries forward, in terms of equitable socio-economic development (9).

The implications of this legal battle therefore may extend far beyond Canada's borders. Canada has one of the strongest legislative protections in the entire world to support equitable access to care by preventing private payment for medically necessary services. Indeed, Canada ranks highest in the world on the UHC Service Coverage Index, at 89 on a scale of 0 to 100, surpassing comparator countries such as Australia, New Zealand, Norway, the UK, the Netherlands, Sweden, and other G-7 nations including the USA, France, Germany, Italy, and Japan (10–12). BC's constitutional challenge threatens to undermine the high bar for healthcare equity that Canada has set for the world. If the corporate plaintiffs are successful in their bid to overturn key provisions of BC's provincial law, then the implication for Canada's other provinces and territories is uncertain given that they all have similar healthcare laws. If, for example, the Canada Health Act itself were to become unenforceable as a consequence of this litigation or subsequent legal challenges, there would be profound implications for the rest of Canada. Globally, although Canada's healthcare system is far from perfect, it is one of the “oldest and most celebrated in the world” (13), recognized for its global leadership on health, and seen as a “beacon of light” (14) for countries aspiring to UHC. Thus, when the trial began on September 6, 2016, the Government of British Columbia entered into a legal battle with Cambie Surgeries Corporation—a private, for-profit, investor-owned corporation—that may shape the future of Canada's healthcare system and influence the design of UHC globally.

## What History Underpins *Cambie Surgeries Corporation v. British Columbia*?

Canadian provincial governments have occasionally faced legal challenges to provincial laws—such as BC's MPA—that parallel the Canada Health Act e.g., *Chaoulli v. Québec* (15) and *Allen v. Alberta* (16). For 37 years, however, the CHA has endured as an effective piece of federal legislation for ensuring equitable access to care, by controlling the growth of private funding for medically necessary care otherwise insured under each of Canada's 13 provincial and territorial publicly-funded Medicare plans. Designed “to protect, promote and restore the physical and mental well-being of residents of Canada and to facilitate reasonable access to health services without financial or other barriers,” the CHA was passed by Parliament in 1984, under the charismatic leadership of then-Health Minister Monique Bégin and Prime Minister Pierre Elliot Trudeau (17).

Although many countries attempt to limit the growth of a private market for physicians' services, the CHA and provincial/territorial laws makes Canada virtually unique among liberal democracies, effectively outlawing physicians enrolled in the public plan from “extra billing” patients directly for insured health services, whether *via* patients' personal out-of-pocket payments or through private duplicative health insurance. The term “effectively” is used because a province or territory may only qualify for the full federal cash contribution, *via* the Canada Health Transfer, if no extra billing occurs. The federal CHA is ingeniously designed to penalize any provincial or territorial government allowing extra billing, by clawing back federal cash and tax-transfers, dollar for dollar, equivalent to the amounts billed in contravention of the Act. Without the federal transfer, most Canadian provinces and territories could not afford to publicly fund their Medicare systems (17). Provincial and territorial governments are, thus, highly incentivized to prevent extra billing (18).

Both federal (CHA) and provincial/territorial restrictions on privately-funded care in Canada worked effectively for 20 years, until a court decision in Québec in 2005 (15). That claim was initially dismissed by both Québec's Superior Court and Québec's Court of Appeal, but these decisions were later overturned in a controversial decision by the Supreme Court of Canada (19). That ruling led to a distinct “watering down” of the CHA's effect, but *only* in Québec. Private duplicative health insurance to cover the costs of three elective procedures (hip and knee replacements and cataract surgeries) was thus made legal in Québec under Bill 33 (20). However, no market for duplicative private insurance has emerged in Québec for these three procedures, mainly because (a) the incentive to develop a commercial insurance market was significantly reduced by new regulations to limit wait times for those procedures, and by enactment of regulations prohibiting, (b) physician dual practice (i.e., being paid by the public purse while also providing privately-funded services that would otherwise be covered under the public plan), and (c) co-mingling of participating (state-funded) physicians and non-participating (privately-funded) physicians in the same surgical facilities (21, 22). The result is that a profitable insurance market is simply not there, (at least not yet). Nevertheless, growth in private investor-owned surgical facilities has followed in Québec, as has growth in the number of Québec's physicians not participating in Medicare. It has been challenging for Québec to enforce these regulations at the public/private interface ever since (23).

Legal scholars have argued about the propriety of the courts' interpretation and application of the Charter of Rights in the Chaoulli case (24, 25). Some experts have criticized that decision as having ignored the empirical health economic and health services research evidence that was adduced in the courtroom, with the judges favoring instead their own views of what healthcare Québec's residents should be able to purchase privately (26).

In May 2007, BC's Medical Services Commission informed CSC that they were concerned about CSC's extra billing of patients. In September 2008, the MSC advised CSC of its intent to audit their records and employ its investigation powers. In January 2009, CSC filed a Writ of Summons against the MSC, in an attempt to outflank the BC Government by legally attacking the constitutionality of BC's law. The plaintiffs' claim is that the MPA infringes Canadians' rights under Canada's constitution, including rights to life, liberty, and security of the person under Section 7 of the Canadian Charter of Rights and Freedoms. In essence, CSC claims that the MPA puts unfair limitations on access to the health services Canadian citizens can purchase privately—particularly where they assert the wait-times for elective surgery in Canada's publicly-funded care system are too long.

## What Are the Contrasting Positions of the Two Parties?

In the assembly of multiple expert opinions sought by counsel for both sides of this newsworthy case (27), there are two opposite points of view, summarized as follows:

**VIEW #1:** *Private health insurance and privately-funded healthcare are “normal” and desirable features of nearly all liberal democracies*' *national health systems, even where the state is providing comprehensive, publicly-funded care for the majority; no serious harm results from such dual systems of care*. The plaintiffs' expert witnesses supporting this view acknowledged that, in most of these dual-systems, public-sector regulators rein in the less-desirable aspects of privately-funded care—such as its impact on equity or its tendency to charge whatever the market will bear—creating an inflationary pressure on physician fees paid in both the public pay and private pay systems (25). In addition, some OECD countries, such as The Netherlands, heavily regulate private health insurance to reduce discriminatory practices, such as risk selection, including refusal of coverage due to pre-existing health conditions. Some of these experts contended that much good accrues to the public care system through the existence of private pay services, such as the purported “steam valve” effect for elective procedures. In their view, the private system siphons off paying patients from the public system, reducing total caseload and overall public expenditures on health, whilst also shortening public system wait-times (28).

**VIEW #2:** *Privately-funded healthcare tends to increase socio-economic and health inequalities in any system that is otherwise publicly-funded and universally accessible. This has adverse consequences for the health status, wellbeing, and productivity of the population, for total care-system cost, and for efficiency of healthcare spending* (Box 1) (29, 30). Expert witnesses for the defendants and the Government of Canada argued that siphoning off selected private-pay, lower-risk, patients, in favor of less complicated—but often higher-priced, high volume—elective procedures has negative consequences for patients and for the operation of the publicly-funded healthcare system. They cite the diversion of scarce medical resources (especially skilled surgeons' and other healthcare providers' time) to the private sector from the public one. The overall result, these experts said, is a fundamental change in who receives timely care—toward care being meted out on the basis of ability to pay, as opposed to clinical need and priority (24–26). These experts were also concerned that allowing privately-funded providers to essentially “charge what the market will bear” could also inflate costs in the public pay system—for example, *via* having to entice surgeons to continue practicing in the public-pay system through higher fees per procedure. [To be fair, some experts have also pointed out that much international evidence on such issues is irrelevant to Canada's virtually unique care system and funding model (31)].

Box 1

**Pan-sectoral effects of increasing inequality.**
- Increased levels of violence, including but not limited to homicide
- Lower economic growth
- Lower levels of child development and educational attainment by adulthood
- Worse psychometric indicators of social capital/cohesion, trust, and civic engagement/societal participation
- Worse levels of population indices combining diverse measures of both health and social problems, including outcomes such as life expectancy, literacy, infant mortality, teen births, obesity, mental illness, imprisonment, social mobility

## What Is the Converging Evidence on the Broader Impacts of Inequality?

Box 1 summarizes the remarkable convergence now occurring, across diverse literatures spanning many research disciplines, concerning the pernicious societal effects of inequality *in and of itself* (29, 30).

This body of evidence suggests that any structural feature of a modern society which fosters increased inequality—including enabling wealthier or more privileged persons to access higher-quality or faster medical care than is available for the majority of citizens—can be expected to lead to worse overall societal outcomes. A wide range of indicators of a healthy, creative, productive, and generally successful society are typically made worse by higher levels of income and social inequality. The increased provision of privately-funded healthcare, in any society where it has been historically tightly regulated (as in Canada) can thus be expected to produce negative impacts on that society. Remarkably, it seems that the mere public perception of unfairness in the provision of healthcare, matters just as much as the reality (29, 30). In settings with long-established, publicly-funded, free-at-point-of-care systems, such as Canada's, it is reasonable to conclude that any significant expansion of privileged care for those who can pay for it privately might well trigger some negative effects beyond the health sector.

In *Cambie Surgeries Corporation v. British Columbia* the court essentially agreed with View #2, as follows:

“[2655] Overall, I find that there is evidence to suggest that duplicative private healthcare would exacerbate wealth and health inequality. I also accept the evidence.that socioeconomic status is a significant determinant of overall health and well-being and poor health status disproportionately affects lower income individuals. Further, duplicative private healthcare and the creation of a two-tier system, where access to preferential treatment would be based on the ability to pay, would exacerbate health inequity in terms of access to healthcare, utilization of healthcare and health outcomes” (1).

## What Are the Broader Implications for Uhc Globally?

The relative merits of these two contrasting points of view were fought out in a Vancouver courtroom when the proceedings commenced in 2016. A legal adjudication process is inherently very different from its closest scientific analog: conducting a rigorous structured systematic review of the empirical evidence (26). The final outcome of the case—whether after the first appeal heard in June 2021, or potentially again at the Supreme Court of Canada—may or may not conform to what the most eminent scholars in the field believe the evidence says. If BC's Medicare Protection Act were to be struck down on appeal, BC would lose what has been a remarkably effective policy by most global standards, opening a crack in Canada's ever-popular publicly-funded Medicare system. The giant global for-profit care and health insurance industry, much of it based just across Canada's shared border with the USA, would be the clear victor. In that event, other countries may want to consider whether they have sufficient safeguards to protect their own UHC from commercial forces likely to increase inequality and inequity through the further global spread of user-pay privately-funded care.

Both health policy experts and international agencies now advocate the extension of UHC to the entire globe. Once a society has achieved widespread UHC (with low or no fees at point of care), it has taken a critical step to invest in its population's long-term health, in the most effective, efficient, and egalitarian manner (4, 5, 32). The dismantling of legal or regulatory controls on privately-funded care, or failure to enforce existing controls, can only be regarded as retrograde, and likely to impede social and economic development—and especially the equity of that development across society.

## Conclusion

We suggest that global health professionals, researchers, policy analysts, scholars, citizenry, and governments familiarize themselves with the scientific and legal aspects of the Cambie trial in Canada, as an exemplary threat to the effective and efficient operation of established and emerging UHC systems globally. Active participation in such debates can constructively support the maintenance and growth of UHC systems as a critical tool in equitable international development. Without such active support, UHC systems could easily fall prey to powerful and wealthy forces worldwide, seeking to make healthcare just another profitable business commodity.

## Data Availability Statement

The original contributions presented in the study are included in the article/supplementary material, further inquiries can be directed to the corresponding author.

## Author Contributions

JF, KSP, CP, CD, and KEP contributed to the conceptualization, analysis, interpretation, drafting, and critical review of the manuscript for important intellectual content. All authors contributed to the article and approved the submitted version.

## Conflict of Interest

JF was retained as an expert witness by counsel for the Attorney-General of Canada in the Cambie trial from 2016–2019. KSP was retained as a consultant/researcher by counsel for the Attorney-General of British Columbia in the Cambie trial. The remaining authors declare that the research was conducted in the absence of any commercial or financial relationships that could be construed as a potential conflict of interest.

## Publisher's Note

All claims expressed in this article are solely those of the authors and do not necessarily represent those of their affiliated organizations, or those of the publisher, the editors and the reviewers. Any product that may be evaluated in this article, or claim that may be made by its manufacturer, is not guaranteed or endorsed by the publisher.

## References

[1] Cambie Surgeries Corporation v. British Columbia (Attorney General). (2020). BCSC 1310. (CanLII). Available online at: https://canlii.ca/t/j9kpw (accessed May 12, 2021).

[2] Cambie Surgeries Corporation v. British Columbia (Attorney General). (2020). BCCA 349 (CanLII). Available online at: https://canlii.ca/t/jc1f9 (accessed May 12, 2021).

[3] Canada Health Act RSC. c C-6 (1985). Available online at: https://canlii.ca/t/532qv (accessed May 12, 2021).

[4] Holmes D. Margaret Chan. Committed to Universal Health Coverage. Lancet. (2012) 380:879. 10.1016/S0140-6736(12)61493-7 22959376 PMC7134571

[5] Lancet (Editorial). The struggle for Universal Health Coverage. Lancet. (2012) 380:859. 10.1016/S0140-6736(12)61485-8 22959368

[6] World Health Organization Geneva Switzerland. WHO/World Bank Convene Ministerial Meeting to Discuss Best Practices for Moving Forward on Universal Health Coverage. (2013). Available online at: https://www.who.int/mediacentre/news/statements/2013/uhc_20130219/en/ (accessed May 12, 2021).

[7] World Bank. Inequality in Focus. Washington, DC: World Bank (2015). Available online at: http://www.worldbank.org/en/topic/isp/publication/inequality-in-focus (accessed May 12, 2021).

[8] The Guardian. (2012). Available online at: https://www.theguardian.com/society/sarah-boseley-global-health/2012/oct/01/worldbank-healthinsurance (accessed May 12, 2021).

[9] United Nations Department of Economic and Social Affairs. (2015). Available online at: https://sdgs.un.org/goals (accessed May 12, 2021).

[10] World Health Organization. The Global Health Observatory, UHC Index of Service Coverage. Available online at: https://www.who.int/data/gho/data/indicators/indicator-details/GHO/uhc-index-of-service-coverage (accessed July 15, 2021).

[11] Index Mundi. UHC Service Coverage Index. Available online at: https://www.indexmundi.com/facts/indicators/SH.UHC.SRVS.CV.XD (accessed July 15, 2021).

[12] World Health Organization. Primary Health Care on the Road to Universal Health Coverage, 2019. Monitoring Report. License: CC BY-NC-SA 3.0 IGO (2021). Available online at: https://apps.who.int/iris/handle/10665/344057 (accessed September 1, 2021).

[13] Martin D Miller AP Quesnel-Vallée A Caron NR Vissandjée B Marchildon GP. Canada's universal health-care system: achieving its potential. Lancet. (2018) 391:P1718–35. 10.1016/S0140-6736(18)30181-8 29483027 PMC7138369

[14] Clark J Horton R. Canada's time to act. Lancet. (2018) 391:1643–5. 10.1016/S0140-6736(18)30176-4 29483020

[15] Chaoulli v. Quebec (Attorney General) [2005] 1 S.C.R. 791. SCC 35 (2005). Available online at: https://scc-csc.lexum.com/scc-csc/scc-csc/en/item/2237/index.do (accessed May 12, 2021).

[16] Allen v. Alberta. ABQB 184 (CanLII). (2014). Available online at: https://canlii.ca/t/g6ddt (accessed May 12, 2021).

[17] Flood CM Archibald T. The illegality of private health care in Canada. Can Med Assoc J. (2001) 164:825–30. Available online at: https://www.cmaj.ca/content/164/6/825 11276552 PMC80881

[18] Palmer KS. Can medical doctors in British Columbia charge patients for hospital and physician care? Infographic Quoi Media. (2021). Available online at: https://quoimedia.com/can-medical-doctors-in-british-columbia-charge-patients-for-hospital-and-physician-care/ (accessed May 12, 2021).

[19] Cambie Surgeries Corporation v. British Columbia (Attorney General). BCSC (1310). (CanLII). (2020). p. 230. Available online at: https://canlii.ca/t/j9kpw#par230 (accessed May 12, 2021).

[20] An Act to amend the Act respecting health services and social services and other legislative provisions SQ 2006. c 43. (2006). Available online at: https://canlii.ca/t/52mz3 (accessed May 12, 2021).

[21] Flood CM Xavier S. Health care rights in Canada: the Chaoulli legacy. Med Law. (2008) 27:617–44. 19004386

[22] Cambie Surgeries Corporation v. British Columbia (Attorney General). BCSC (1310). (CanLII). (2020). p. 2589. Available online at: https://canlii.ca/t/j9kpw#par2589 (accessed August 31, 2021).

[23] Cambie Surgeries Corporation v. British Columbia (Attorney General). BCSC (1310). (CanLII). (2020). p. 2245. Available online at: https://canlii.ca/t/j9kpw#par2245 (accessed May 12, 2021).

[24] Flood CM Stabile M Kontic S. Finding health policy “arbitrary”: the evidence on waiting, dying and two-tier systems. In: Flood CM editor. Access to Care, Access to Justice: The Legal Debate over Private Health Insurance in Canada. Toronto, ON: University of Toronto Press (2006). p. 296–300.

[25] Flood CM Haugan A. Is Canada odd? A comparison of European and Canadian approaches to choice and regulation of the public/private divide in health care. Health Econ Policy L. (2010) 5:319–41. 10.1017/S1744133110000046 20602857

[26] Wright CG. Different interpretations of ‘evidence’ and implications for the Canadian healthcare system. In: Flood CM editor. Access to Care, Access to Justice: The Legal Debate over Private Health Insurance in Canada. Toronto, ON: University of Toronto Press (2006). p. 259–72.

[27] Day B. Health Care Hampered by Red Tape - excerpted from Vancouver Sun. (2016). Available online at: https://www.brianday.ca/news/hampered-by-red-tape/ (accessed May 12, 2021).

[28] Siciliani L Hurst J. Tackling excessive waiting times for elective surgery: a comparative analysis of policies in 12 OECD countries. Health Policy. (2005) 72:201–15. 10.1016/j.healthpol.2004.07.003 15802155

[29] Wilkinson RG Pickett KE. The Spirit Level: Why Equality is Better for Everyone. London: Penguin (2010).

[30] Pickett KE Wilkinson RG. Income inequality and health: a causal review. Soc Sci Med. (2015) 28:316–26. 10.1016/j.socscimed.2014.12.031 25577953

[31] Currie G Donaldson C Lu M. What does Canada profit from the for-profit debate on healthcare? Can Public Pol. (2003) 29:227–35. 10.2307/3552457

[32] Marmot M. Universal Health Coverage and social determinants of health. Lancet. (2013) 382:1227–8. 10.1016/S0140-6736(13)61791-2 24120189

